# Supplementary material for: A New Natural Defense Against Airborne Pathogens
Source: QRB Discov. 2020 Jul 7;1:e5. doi: 10.1017/qrd.2020.9 (PMC7453358; doi:10.1017/qrd.2020.9)
Supplement: Supplementary file 1 [file qrdsup.zip › S2633289220000095sup001.pdf]

SUPPLEMENTARY MATERIALS FOR

A New Natural Defense Against Airborne Pathogens

David Edwards (1,7),* Dennis Ausiello (2,3), Anthony Hickey (6), Richard Batycky (8), Lester Griel (5), Michael Lipp (8), Wes Dehaan (9), Robert Clarke (11), David Hava (11), Jason Perry (11), Brendan Laurenzi (11), Aidan K. Curran (11), Brandon J. Beddingfield (10), Chad Roy (10), Robert Langer (4)

*** Corresponding author**

This PDF contains the following:

- Methods: Mucus Mimetic Studies
- Human Phase I Clinical Study Adverse Event Summary
- Human Safety Margin
- Exhaled Bioaerosol Data
- Nimbus Characteristics
- Gene Expression
- Figure S1. Change of expired bioaerosol at the low clinical trial dose
- Figure S2. Change of expired bioaerosol at the moderate clinical trial dose.
- Fig. S3 Nasal spray versus Nimbus size distribution
- Table S1 Fold Change of 19 genes following FEND treatment

Methods: Mucus Mimetic Studies

For delivering bacterial and viral pathogens onto mucus mimetic stock solutions of FEND formulations were made by dissolving calcium chloride in sodium chloride with a varying molar ratios of Ca2+ to Na+. Liquid formulations were nebulized into the chamber using an in house developed sedimentation chamber and allowed to settle by gravity over a 5 minute period. After the exposure of the mucus mimetic to the indicated formulations, 10µL of bacterial suspension (1:10 dilution of an OD600 ~0.3 suspension) or 50 µL of undiluted Influenza A/WSN/33/1 (~ 106.5 TCID50/well) or Rhinovirus (Rv16; 107.6 TCID50/mL) was added to apical surface of sodium alginate and 0.5-1.0mL of sterile isotonic saline was added to the basolateral chamber of the Transwell. At different time points after the addition of bacteria or virus to the apical surface, a sample of the basolateral buffer was collected. The number of bacteria was determined by serial dilution and plating 100µL of each dilution on appropriate growth media and incubation overnight at 37°C and 5% CO2 for enumeration of colony forming units. Viral titer was determined by PCR or TCID50 using HeLa cells.

Table 12.2.1:1Incidence of Treatment-Emergent AEs

Treatment (No. of Subjects Dosed)	Number of Subjects Reporting AEs	Number of AE Episodes Reported
12.9 mg FEND1 (8)	4(50%)	6
38.7 mg FEND1 (8)	4(50%)	5
77.4 mg FEND1 (8)	3(38%)	6
Placebo (8)	0 (0)	0
Total	7 (88%)	17

Adverse Events: Human Phase I Study FEND

1. Brief Summary of Adverse Events

Of the 8 subjects participating in this clinical trial, all 8 completed all 4 escalating dose treatment days. Thus, no subject was discontinued due to an adverse event (AE), and similarly there were no serious AEs (SAEs) reported in this. A total of 17 TEAEs were reported by 7 (88%) of the 8

subjects dosed in this study, with 4 (50%) subjects following 12.9 mg FEND1, 4 (50%) subjects following 38.7 mg PUR003, and 3 (38%) subjects following 77.4 mg FEND1. Of the 17 AEs, 16 were reported as mild in severity and 1 was reported as moderate. The Investigator considered 12 AEs to be improbably related to the study drug and 5 AEs to be possibly related. The following table summarizes the incidence of TEAEs in this study.

Cough was the most commonly reported AE in this study, and was reported a total of 5 times by 4 (50%) subjects, with 2 subjects following 12.9 mg FEND1 reporting 1 episode of cough each (Subjects 4, and 6); and 2 subjects following 77.4 mg FEND1 of which 2 episodes were reported by 1 subject (Subject 1), and the remaining episode was reported by 1 subject (Subject 7). Four episodes were reported as mild in severity with 1 episode reported as moderate. One (1) subject had the nebulizer stopped for approximately 4 minutes during dosing due to coughing, which was assessed as a moderate event. Onset for coughs ranged from 6 minutes to approximately 2.5 hours postdose, and duration ranged from 15 seconds (data obtained from source AE report) to approximately 5.75 days. Subject 4's cough AE coincided with an AE of mild pharyngolaryngeal pain following 12.9 mg FEND1. Administration of 77.4 mg FEND1 for Subject 1 via nebulizer was stopped and re-started due to coughing. The Investigator considered 4 cough episodes (following 12.9 mg and 77.4 mg FEND1) to be possibly related to the study treatment and 1 episode (following 77.4 mg FEND1) to be improbably related to the study treatment.

Mild dizziness was reported a total of 4 times by 4 (50%) subjects, with 3 subjects following 38.7 mg PUR003 and 1 subject following 12.9 mg FEND1. Onset ranged from 1 to 29 minutes postdose. Duration ranged from 4 minutes to approximately 1.5 hours. The Investigator considered all dizziness episodes to be improbably related to the study treatment. No vital sign recordings were performed during these dizziness events.

Mild nausea was reported a total of 4 times by 2 (25%) subjects, with 1 subject following both 38.7 mg and 77.4 mg FEND1, (2 separate events of nausea followed this dose), and 1 subject following 12.9 mg FEND1. Onset ranged from 1 minute to approximately 4 hours postdose. Duration ranged from 7 minutes to 2 hours. The Investigator considered all nausea episodes to be improbably related to the study treatment.

Mild fatigue was reported a total of 3 times by 3 (38%) subjects, with 1 subject each following 12.9 mg, 38.7 mg, and 77.4 mg FEND1. Onset ranged from approximately

2.5 hours to approximately 1 day postdose. Duration ranged from approximately 3 hours to 20 hours. The Investigator considered all fatigue episodes to be improbably related to the study treatment.

No concomitant medications were administered for AEs in this study.

Safety Summary

A total of 17 TEAEs were reported by 7 (88%) of the 8 subjects dosed in this study, with 4 (50%) subjects following 12.9 mg FEND1, 4 (50%) subjects following 38.7 mg FEND1, and 3 (38%) subjects following 77.4 mg FEND1. Of the 17 AEs, 16 were reported as mild in severity and 1 was reported as moderate. The Investigator considered 12 AEs to be improbably related to the study drug and 5 AEs to be possibly related. The most commonly reported AEs were cough and dizziness.

OVERALL CONCLUSIONS

There were no SAEs reported in this study and none of the subjects were discontinued due to an AE. AE incidence was similar across the active treatment groups with AE number and type being comparable. Therefore, a treatment-related trend was observed, as no subjects reported AEs after receiving placebo treatment. Human Safety Margins

Human Safety Margins

Safety margins for human exposure to inhaled products are calculated from animal studies with the assumption that the majority of the inhaled aerosol in animal studies is deposited in the nose due to the obligate nasal breathing and complex nature of rat and especially dog nasal turbinate structures. The percent of aerosol delivered to the nose of these animals that is considered to reach the lungs is 10% in the rat and 25% in the dog. Conversely, for our nasal safety margin calculations, we have assumed the reverse, namely that the nasal deposition of the delivered aerosol in the animal studies is 90% of the delivered dose in the rat and 75% in the dog. Based on the achieved NOAELs in the rat and dog inhalation studies, and a proposed $CaCl_2$ delivered dose in humans of 2 mg, the calculated safety margins for inhaled delivery for FEND 1 and FEND 2 in the rat are >150-fold and >110-fold respectively. In the dog, the inhaled safety margins are >70-fold and >150-fold. For nasal delivery of FEND 1 and FEND 2, the safety

margins are >1200-fold and >220-fold in the rat respectively. In both cases, the nasal safety margin derived from the dog data are <1-fold. This is due to the extremely large surface area of the dog relative to its size (220 cm^2 for a 10kg average dog relative to 181 cm^2 for a 60kg average human). Accordingly, the dose needed for a dog to achieve a reasonable nasal safety margin would be unachievable. Given the very substantial margins for inhalation in the dog and the large margins for the rat for both nasal and inhaled doses, as well as the absence of any nasal findings in either species, or nasal adverse findings in clinical studies, we believe that the data are clear that nasal application nasal application of FEND formulations will be safe and well tolerated.

Exhaled Bioaerosol Data

A total of eight (n=8) human subjects were administered ascending pulmonary doses of FEND1 (12.9 mg, 38.7 mg, and 77.4 mg $CaCl_2$), in the target efficacy range of 0.25 mg $CaCl_2$/kg to 1.0 mg/$CaCl_2$/kg. Suppression of exhaled bioaerosol was pronounced at the highest dose, while at the low dose (Figure S1) and intermediate dose (Figure S2) no systematic variation was observed between the active and placebo control.

Nimbus Characteristics

The hand-held nebulizer (Nimbus) operates on the basis of a vibrating mesh activated by two replaceable AAA batteries. The device is comprised of a *head*, which contains the piezoelectric vibrating mesh and on/off trigger, and a *base* or 1 oz (30 mL) vial into which the FEND solution can be filled. The Nimbus vial is detachable and made either of glass or plastic, full of sterile solution and discarded once empty. To evaluate delivered dose a 4-place balance (0.1 mg precision) was used along with the hand-held nebulizer. Nimbus was inverted and cloud dispensed into a 6 ounce jar covered by a disk with a hole for cloud emission into the glass container. After ten seconds the cloud ceased to form, the Nimbus was removed, the disk removed, and the weight of the glass determined. The "Discharged Dose" (n=5) results comprise measuring the entire 10-second emission into the jar through the coaster, and capping the coaster hole immediately after. The total emitted mass from the device was determined to be 57.0 ± 2.1 mg. Approximately 22.1 ± 1.5 of the dose deposited on the walls of the glass or ~39% of the emitted dose. Nasally delivered dose was assessed by two users affecting a single nasal inhalation from the glass post filling (n=5). The results 22.6 mg and 23.4 mg, respectively suggest a reproducible delivery of the solution and in the range of the target nasal dose.

Figures S3 illustrates the particle size distribution from the Nimbus versus the nasal spray.

Gene Expression

We evaluated mRNA expression of 26 genes following application of $CaCl_2$ formulations compositions (Supplementary Material). We found that seven of the 26 genes consistently expressed 3-fold or greater expression after treatment. Table S1 summarizes the fold change of the other 19 genes.

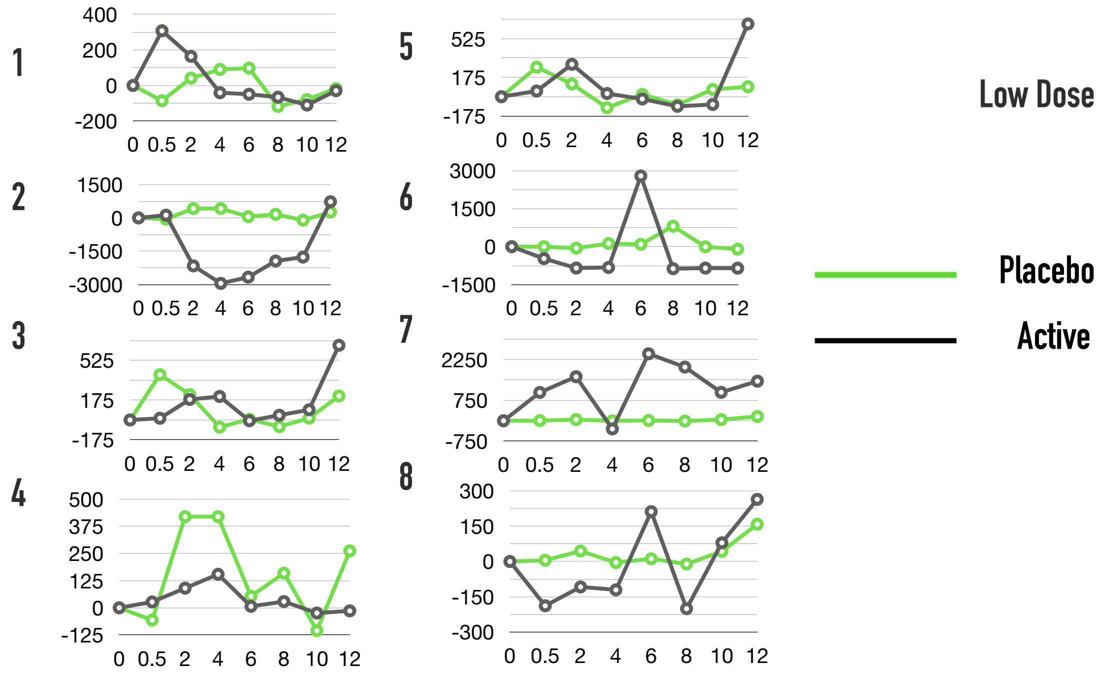

Figure S1. Change of expired bioaerosol from human lungs following treatment by FEND1 at the low clinical trial dose. Exhaled bioparticles per liter versus hours post dosing (relative to baseline) of the 8 human subjects following FEND1 treatment or following saline placebo control. Bioparticle sampling from exhaled air was performed at the following time points: prior to dosing and at 0.5, 1, 2, 4, 6, 8, 10, and 12 hours post-dose for each period, and prior to release from the clinic on Period 4, Day 2. Each subject was treated with a placebo (saline) control and with each of the three doses of FEND1 at 24h intervals. Data from each subject's high dose recording are compared with each subject's placebo control.

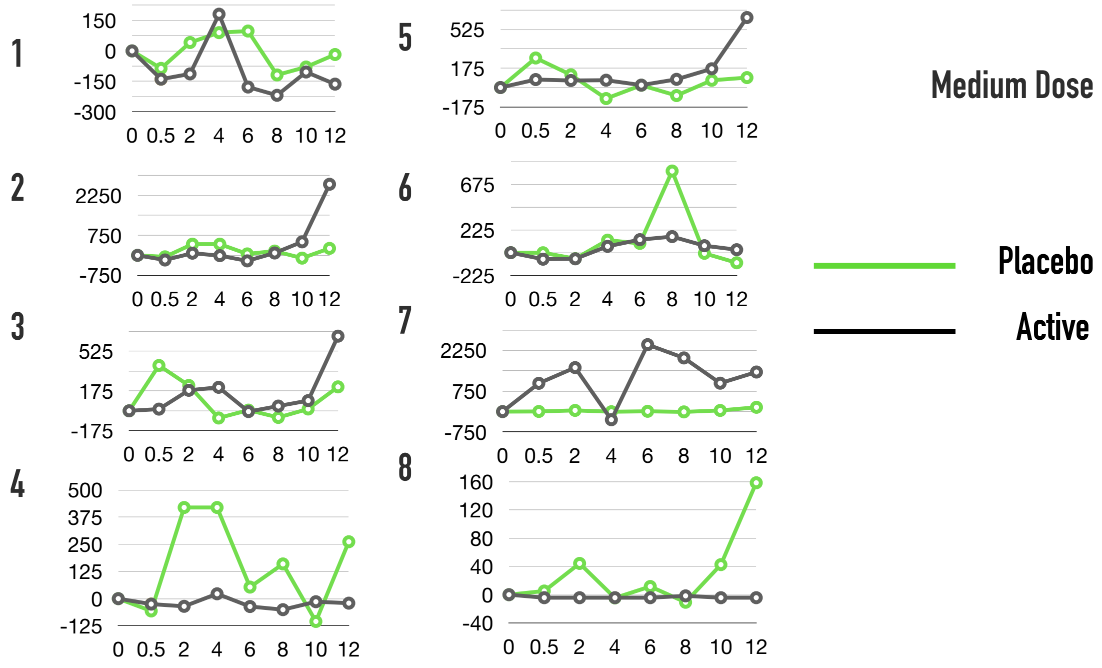

Figure S2. Change of expired bioaerosol from human lungs following treatment by FEND1 at the moderate clinical trial dose. Exhaled bioparticles per liter versus hours post dosing (relative to baseline) of the 8 human subjects following FEND1 treatment or following saline placebo control. Bioparticle sampling from exhaled air was performed at the following time points: prior to dosing and at 0.5, 1, 2, 4, 6, 8, 10, and 12 hours post-dose for each period, and prior to release from the clinic on Period 4, Day 2. Each subject was treated with a placebo (saline) control and with each of the three doses of FEND1 at 24h intervals. Data from each subject's high dose recording are compared with each subject's placebo control.

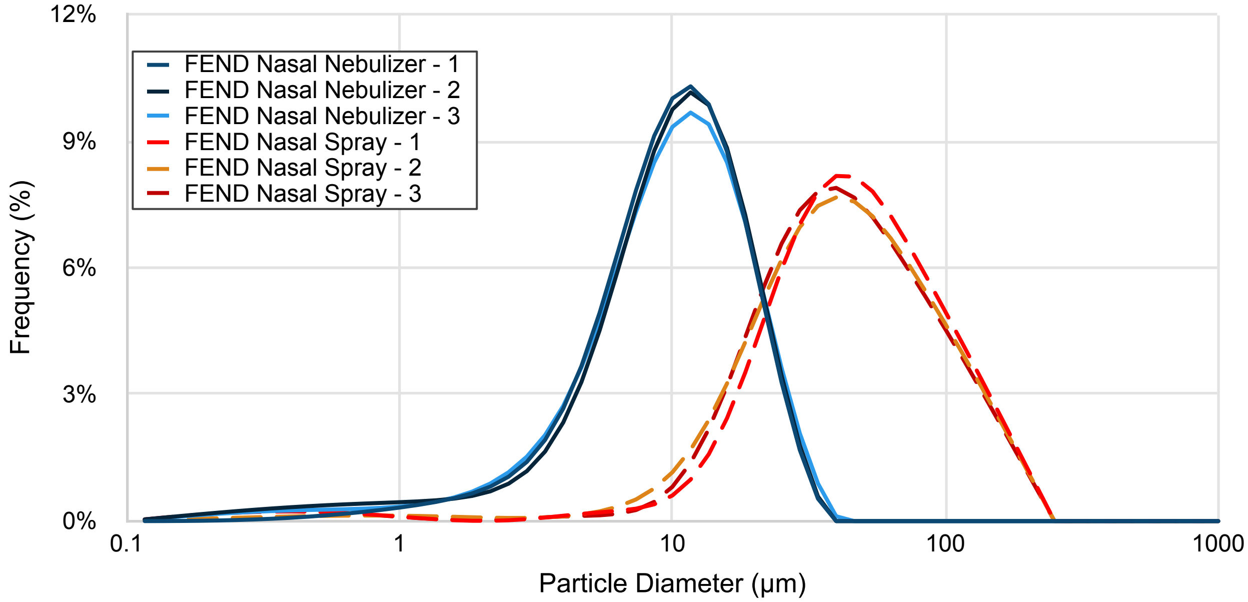

Figure S3. Comparison of hand-held nebulizer and nasal spray particle size distributions. An open-beam laser diffraction system (Malvern Spraytec) assessed geometric size distributions of emitted droplets from Nimbus (Figure 5a) and the nasal spray (Figure 5b). The two populations of particles reflect two deposition patterns in the nose, with the nasal spray (red) profile projecting an anterior (frontal) deposition in the nose and the nebulizer a more distributed deposition from anterior to posterior of the nose.

Donor	042D		040D		035E		019E		023E		018E	
	2X	8X	2X	8X	2X	8X	2X	8X	2X	8X	2X	8X
BD-2	NT	73.07	3.645	120.8	23.71	144.4	45.68	6.851	3.13	430.5	4.471	21.71
IL-6	NT	47.43	2.544	18.18	6.011	27.05	20.42	3.213	1.724	60.06	2.087	2.924
IL-8	NT	24.98	3.545	28.253	2.595	25.85	15.72	2.142	1.29	39.44	1.784	4.429
IP-10	NT	2.336	5.976	9.591	2.484	9.253	ND	-2.242	ND	ND	1.536	3.37
MUC5AC	NT	4.217	1.062	3.313	1.353	2.171	2.839	1.278	2.6	18.67	2.004	3.148
MUC5B	NT	6.138	2.167	3.699	2.386	3.812	3.535	2.513	1.926	11.74	3.346	4.484
RANTES	NT	28.01	-11.11	-1.497	19.48	5.407	1.387	-2.207	1.121	-6.438	-1.904	-2.875
αENac	NT	-1.732	-1.734	-2.140	-1.448	-1.719	-1.946	-1.585	-1.084	-1.294	-1.143	-1.685
B2m	NT	N/A	NT	NT	NT	NT	NT	NT	NT	NT	NT	NT
B-actin	NT	N/A	NT	NT	NT	NT	NT	NT	NT	NT	NT	NT
BD-1	NT	2.124	-1.847	1.673	1.533	2.25	1.925	1.594	1.024	-1.593	-1.215	-1.620
BD-3	NT	NT	NT	NT	NT	NT	NT	NT	ND	ND	ND	NQ
CFTR	NT	ND	NT	NT	ND	ND	NT	NT	ND	ND	ND	ND
GAPDH	NT	N/A	N/A	N/A	N/A	N/A	NT	NT	N/A	N/A	N/A	N/A
ICAM-1	NT	NT	NT	NT	NT	NT	NT	NT	ND	ND	NT	NT
INFα	NT	NT	NT	NT	ND	ND	NT	NT	-1.146	2.698	ND	ND
INFβ	NT	NT	NT	NT	ND	NQ	NT	NT	ND	ND	ND	NQ
LL-37	NT	ND	NT	NT	1.517	ND	NT	NT	1.527	0.6775	-4.048	-3.923
MCP-1	NT	20.09	-1.229	-1.135	NQ	NQ	-2.006	-1.793	NQ	NQ	-2.069	-1.903
MMP-9	NT	NT	NT	NT	72.59	97.53	NT	NT	1.088	0.0451	-2.122	-1.754
SP-A1	NT	12.39	ND	ND	1.218	-1.41	-1.055	-1.571	-1.5	-2.294	-2.604	-2.076
SP-A2	NT	NQ	NT	NT	NQ	NQ	NT	NT	NQ	NQ	NQ	NQ
SP-C	NT	NQ	NT	NT	NQ	NQ	NT	NT	NQ	NQ	NQ	NQ
SP-D	NT	NQ	NT	NT	NQ	NQ	NT	NT	NQ	NQ	NQ	NQ
TNFα	NT	NT	NT	NT	ND	ND	NT	NT	ND	ND	ND	ND
TRAIL	NT	-1.341	1.269	1.572	1.11	-1.059	-1.017	-1.176	-1.088	-1.169	-1.215	-1.62

NT: Not Tested, ND: Not Detected, N/A: Assayed but no fold change values available, NQ: Not Quantifiable

Table S1. Fold Change of 19 genes following FEND treatment following 24-hours post treatment. Targets showing multiple products in their melt curve analysis were marked as NQ. Targets with Ct values > 36 were marked as NT. Targets lacking gene expression values are listed as NT. Housekeeping genes were not analyzed N/A.
